# Cognitive Reserve as a Predictor of Cognitive Decline, but Not Age of Diagnosis in Patients with Young‐Onset Alzheimer's Disease: An Underexplored Population

**DOI:** 10.1002/alz70858_098895

**Published:** 2025-12-25

**Authors:** Anat Marmor, Zeev Meiner, Shlomzion Kahana Merhavi, Eli Vakil

**Affiliations:** ^1^ Bar Ilan University, Ramat Gan, Israel, Israel; ^2^ Hadassah Hebrew University Medical Center, Jerusalem, Israel, Israel; ^3^ Hadassah Hebrew University Medical Center, Jerusalem, 97654, Israel

## Abstract

**Background:**

The cognitive reserve (CR) theory posits that higher CR can delay Alzheimer's onset and influence disease progression. While this has been studied in late‐onset Alzheimer's disease (LOAD), its effects in young‐onset Alzheimer's disease (YOAD, onset before age 65) have not been extensively explored. This study aims to examine whether high CR in YOAD delays onset and leads to faster cognitive deterioration, as seen in LOAD.

**Methods:**

This retrospective study included 72 patients (39 females, age range: 46‐64 years) diagnosed with YOAD, selected from a memory clinic database of 2,040 dementia patients. These patients were followed for three years, with cognitive function assessed using the Mini‐Mental State Examination (MMSE).

**Result:**

Age of diagnosis did not correlate significantly with CR variables, years of education or family size. However, education, family size, and gender predicted cognitive decline six months after diagnosis. Higher education was associated with greater decline, larger families with lower decline. Women showed higher decline rates than men.

**Conclusion:**

In contrast to studies among LOAD, there was no significant correlation between age and CR among YOAD, suggesting that other mechanisms might be more influential than CR parameters in younger individuals. However, similar to LOAD, YOAD patients with higher education and smaller families experienced faster disease progression, implying that diagnoses are frequently made when brain pathology is already severe. These findings highlight the urgent need for early detection tools and cognitive interventions to ease the challenges faced by these young patients.

